# Editorial: Trends in Optical/Laser Spectroscopy and Applications

**DOI:** 10.3390/molecules26071929

**Published:** 2021-03-30

**Authors:** Mihail Lucian Pascu

**Affiliations:** 1 Laser Department, National Institute for Laser, Plasma and Radiation Physics, 405 Atomistilor Street, P.P.BOX: MG-36, RO-077125 Magurele, Romania; mihai.pascu@inflpr.ro; Tel.: +40-21-457-4524; 2 Faculty of Physics, University of Bucharest, RO-077125 Magurele, Romania

Optics and optical spectroscopy are dynamic fields that are developing very fast nowadays, triggered by (i) the need to go deeper in the scientific approach to nature’s processes and phenomena, (ii) the evolution of applications in technological and industrial processes, art conservation, environment protection and cosmic space, and (iii) the sometimes hard to predict evolutions of knowledge in science, life sciences, artistic culture, technology and industrial processes.

Optical spectroscopy works in the visual spectral range of electromagnetic radiation covering, as is generally agreed, the domain extending between 100 nm and 1÷1.2 µm. It uses either optical sources of incoherent radiation (lamps, bulbs, sun, moon, stars, outer space emissions, earth phenomena etc.) or laser sources that emit coherent radiation with very well-known characteristics. 

Historically, spectroscopy was systematically approached as early as the 17th century by Newton’s observations of colours in white light and the colour distribution of sunlight [1,2]. The first observations were dedicated to measurements and characterization of light emitted by natural sources, as well as to the absorption of this light by surrounding media. In the early stages, the most frequent studies were dedicated to the light absorption of liquid samples, which led to new knowledge about the optical properties of molecules in liquids; in doing this, the studied media were bulky (volumes ranging from millilitres to litres), and the light sources were incoherent along the covered spectral range. Absorption experiments on gas samples, which were developed afterwards, led firstly to knowledge of new molecular as well as atomic components of gaseous states. At later stages, light absorption measurements were used to identify inner energetical levels of molecules and atoms that are included in all kinds of materials, regardless of their aggregate state and macroscopical positions.

Emission spectroscopy was approached in direct connection with absorption studies, but its development slope was higher beginning in the middle of the 19th century in connection with the increasing interest in plasma physics, which at that time was cold plasma only.

Starting from these early steps in its evolution, in the 20th century spectroscopy was diversified, so that today a large area in basic science and applications is covered. This was possible due to the use of lasers and tunable lasers, beginning in the 1950s. One achievement that has been made is the study of certain nuclear properties by means of very high optical resolution. Another domain is laser-induced fluorescence, which combines absorption and fluorescence spectroscopy based on earlier studies with incoherent light sources. Further, Raman spectroscopy was developed, which does not imply absorption and/or emission of light and is based on the inelastic scattering of photons on targets, which is accompanied by a transfer of energy that leads to shifts in the incident beam wavelength. This also gives information about the molecular structure of the target.

The evolution of optical spectroscopy and, in particular, of laser spectroscopy has given rise to progress not only in basic science but in a large panoply of applications as well. In essence, the spectroscopical methods initially developed as basic science tools are now used as omnipotent instruments adapted for applications in technology, engineering, biomedicine, environmental protection, art and many other fields with immediate practical use. 

Nowadays, laser spectroscopy is evolving spectacularly, and new trends may be identified at the levels of basic research and applications, distributed along the following main directions: 

(i) Development of lasers with active media of very small dimensions (submicrometres to nanometres). This is complementary, at the opposite geometrical scale, to the laser physics that uses extremely large laser systems emitting tens of Petawatt (PW, 1PW = 10^15^ Watt) laser beams [3]. A typical laser in this category is the microlitre droplet laser, which uses as its active medium a droplet of liquid optically pumped with another laser beam [4,5,6,7]. Since a laser beam is used for droplet pumping, a controlled match should be made of the beam power (i.e., laser beam pressure) with the absorption of the droplet’s material (i.e., absorbent concentration) [8].

(ii) Performance of spectroscopy experiments (absorption, laser-induced fluorescence, Raman) in which the studied target has very small dimensions, close to the focus spot of a laser beam. This approach is more convenient than the use of larger targets with volumes in the order of some millilitres, where the interaction with the laser/optical incident beam takes place mainly in the focus of it, and the emission is buffered by molecules existing outside the interaction volume. On the contrary, in droplets the emitted signals are available for measurements without the influence/contribution of the buffer, and they keep the information obtained from the focus point better [4,5,9]; this is further enhanced by introducing nanoparticles into the droplet’s content [10].

(iii) Laser spectroscopy of foams and emulsions in bulk or droplets as a direct link with microfluidics and optofluidics [11]. This opens up new and promising applications in biomedical treatments, separation of raw materials and environmental monitoring [12].

(iv) Combination of spectroscopy methods, including molecular docking, with medical treatments to fight multiple drug resistance acquired by bacteria, and illnesses occurring accidentally that are produced by microorganisms [13].

This volume is designed to present new reports about multidisciplinary connections of optics and optical/laser spectroscopy with science, technology and applications in an extended number of fields, from experiments on Earth to outer space working conditions, from medical treatments to environmental protection, and from microfluidics to bioinformatics. It also includes reports in complementary fields such as high-resolution microscopy, biomolecular spectroscopy, fighting multiple drug resistance, optics of miniaturized systems, opto- and microfluidics, spectroscopy of very small volume samples, micro- and nanospectroscopy, and microlasers.

## Data Availability

Data available on request from the authors.
